# Sustainability Evaluation of Pastoral Livestock Systems

**DOI:** 10.3390/ani13081335

**Published:** 2023-04-13

**Authors:** Mohamed Ouali, Fathi Abdellatif Belhouadjeb, Walid Soufan, Hail Z. Rihan

**Affiliations:** 1 Faculty of Natural Sciences and Life, University “Ziane Achour” of Djelfa, Djelfa 17000, Algeria; 2 Centre de Recherche en Agropastoralisme (CRAPAST), Djelfa 17000, Algeria; belhouadjebfathi@gmail.com; 3 Plant Production Department, College of Food and Agriculture Sciences, King Saud University, Riyadh 11451, Saudi Arabia; 4 School of Biological Sciences, Faculty of Science and Environment, University of Plymouth, Drake Circus, Plymouth PL4 8AA, UK

**Keywords:** sheep production, steppe area, sustainability, livestock production systems, fodder production, pastoral

## Abstract

**Simple Summary:**

In order to manage important transformations affecting a steppe area, it is necessary to analyze the existing pastoral system by evaluating the sustainability of its subsystems of production. For this reason, in this study, a tool for the evaluation of the sustainability of livestock production in a steppe area was used to identify the most sustainable systems among the various ways that livestock are managed and produced. The study was conducted in the region ranked first in terms of sheep production. Using a grid for evaluating the sustainability of livestock systems in steppe regions, the impact of each system on the environment (environmental, economic, and social) was examined, and the results showed that the feed system was unbalanced, with high pressure on steppe rangelands. Nevertheless, multiple ways of improving these systems emerged from the analysis, such as encouraging the production of fodder and its association with livestock on new spatial, temporal, regional, and national levels.

**Abstract:**

In order to manage important transformations affecting a steppe area, it is necessary to analyze the existing pastoral system by evaluating the sustainability of its subsystems of production. For this reason, in this study, a tool for the evaluation of the sustainability of livestock production in the steppe area was used in order to identify the most sustainable systems. The study was conducted using a survey of 87 livestock farmers (production units) in the region ranked first in terms of sheep production. Principal component analysis (PCA) enabled us to identify two production systems: (i) the pastoral production system, characterized by the mobility of livestock and its high dependence on concentrated feed; (ii) the agropastoral system, combining fodder and livestock production, which is sedentary and semi-extensive. Using a grid for evaluating the sustainability of livestock systems in steppe regions, the impact of each system on the environment (environmental, economic, and social) was examined, and the results showed that the feed system was unbalanced, with high pressure on steppe rangelands. Nevertheless, multiple ways of improving these systems emerged from the analysis, such as encouraging the production of fodder and its association with livestock, on new spatial, temporal, regional, and national levels.

## 1. Introduction

Pastoralism is defined as rangeland management for extensive livestock production using commonly owned pastures located mainly in arid and semi-arid areas [1]. The Algerian steppe, as a buffer zone between the Tellian chains to the north and the Sahara Desert to the south, is a pastoral region. Sheep farming is the major agricultural practice of the local population of the steppe. It is the homeland of 80% of the national sheep flock, numbering 29.428 million heads in 2019 [2]. Anthropogenic activities, such as the extension of clearings at the expense of rangelands, poor management of water sources and soil [3,4,5,6,7,8], and the frequent drought waves of the last two decades [9,10,11,12,13], have sent steppe plant formations into an extremely worrying phase of degradation. The implemented measures to mitigate the degradation of the steppe have been subject to several studies investigating their effect on the pastoral value, biodiversity, and soil properties [7,8,14,15,16,17,18]; the pastoral system is inseparable from the steppe region [19], and it has been constantly developed in order to adapt to the environmental, economic and social changes. Indeed, “farmers and breeders around the world have accumulated experience and local knowledge that can help them in their adaptation, but the fast change in many farming systems in developing countries may be more than their capacity” [20]. This difficulty to adapt has worsened the sustainability of Algerian steppe ecosystems, and the degradation of natural environments in arid and semi-arid regions has dramatically increased during the last decades [21]. This increase results from the globalization of environmental, economic, and social challenges, which have caused a rapid change in the world of agriculture and rural communities. These challenges include, in particular, the future of extensive livestock systems, facing the expected increase in livestock production, and preserving the environment [22]. In order to understand the major transformations affecting the steppe area, it is, therefore, necessary to analyze the existing pastoral system by evaluating the sustainability of the various subsystems of production. Although many frameworks emphasize the necessity of including socio-economic and environmental aspects in sustainability assessment, many others focus only on environmental indicators to investigate the short- and long-term effects of different agricultural management practices [23]. Indicator-based sustainability assessment frameworks combining environmental, economic, and social issues require the processing of a wide range of information (qualitative and quantitative), parameters, and uncertainties [24]. Among these, the IDEA (“Indicateurs de Durabilité des Exploitations Agricoles“ = “Farm Sustainability Indicators”) method [25,26,27] is one of the widely used sustainability assessment methods in the European Union [28]. This method is capable of observing differences in sustainability between production systems, and it is easy to adapt the method to local context and specific agriculture. This study aimed to highlight the different pastoral systems existing in the region using a typology and to determine the sustainability of the farms according to the different management systems. Therefore, this research contributes to solving a global problem by analyzing the influence of pastoral livestock on the sustainability of steppe ecosystems.

## 2. Materials and Methods

### 2.1. The Study Area

**Figure 1:**
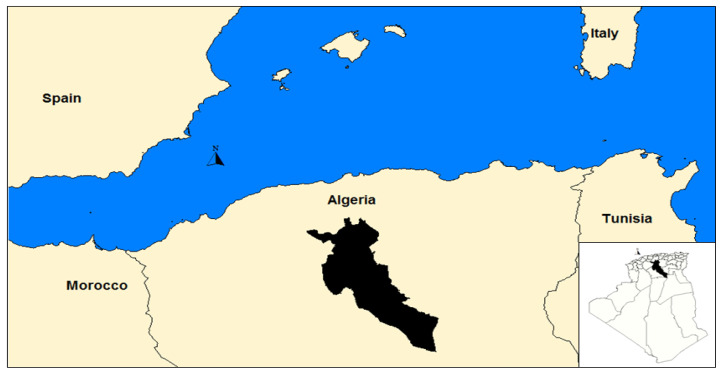
The study area (Djelfa), data source: own illustration.

To conduct this study, the central area of the Algerian steppe was chosen, in particular, the province of Djelfa, as natural pasturelands constitute more than 70% of the overall area of the province [29], where sheep production has been the principal output of these areas. The province of Djelfa ranks first in terms of sheep production in Algeria, with a herd of 3,242,760 heads, representing nearly 12% of the total national sheep herd [30]. The geographical location and a large number of sheep give a better representation of the extensive pastoralism in the Algerian steppe, which is still very present (Figure 1).

### 2.2. Data Collection and Analysis

A total of 87 livestock farms were chosen randomly and according to the cooperation of the farmers regarding the aims of the study. In order to collect the data for the typology (Table 1) and the sustainability evaluation (Table 2), a questionnaire survey was conducted with the owners of the farms (stockbreeders), which was planned for a period of 4 years (until January 2021).

The typology was based on the characteristics and practices typically found on livestock farms of the Algerian steppe. These include both functional and structural practices, covering the multiple facets of pastoral livestock farms, from production to marketing. The table below (Table 1) summarizes the characteristics of differentiation and the related variables.

Sixteen selected variables were analyzed, using hierarchical ascending classification (HAC) and principal component analysis (PCA) to classify the various production systems. In order to evaluate the sustainability of farms based on the different methods and strategies used in management, a grid was developed to evaluate the sustainability of livestock farms in steppe regions. This was mainly based on the IDEA (“Indicateurs de Durabilité des Exploitations Agricoles“ = “Farm Sustainability Indicators”), which is a method that simultaneously evaluates the three levels of sustainability (agro-ecological, economic, and socio-territorial) [31,32,33,34,35,36,37,38,39,40,41,42], defined as a tool to assess the sustainability of a farm. It is multidimensional and serves as a decision aid; however, some modifications and adaptations were made in order to optimize the indicators to the context of sheep farms in the Algerian steppe (Table 2) without changing the components of each scale, nor their annotations. For example, for the indicator “Valuation of patrimony”, in our case the traditional tents and other weaving products represent a perfect model of adaptation to the environment and constitute a method of enhancing the value of the by-products of the livestock activity (wool), as well as creating employment, especially for women, who thus contribute to improving and increasing the household income.

The method of evaluation developed and used includes three scales, i.e., agroecological, economic, and socio-territorial, each of them having several components and each component having its appropriate indicators. The sum of the scores of these indicators constitutes the score of the component, and the sum of the scores of the components constitutes the score of the scale, which is scored on one hundred points (100 points) (Table 2).

According to the same concept as IDEA, the overall sustainability score of the farm is that of the constraining dimension (the lowest score of the three dimensions). This approach makes it possible to have simultaneity in the three dimensions and, therefore, an integrated approach to sustainability [43]. This evaluation is focused on (i) the comparison between different types of pastoral management systems and (ii) the identification of factors that promote and affect the sustainability of the different management systems.

## 3. Results

### 3.1. Typology of Exploitations

**Figure 2:**
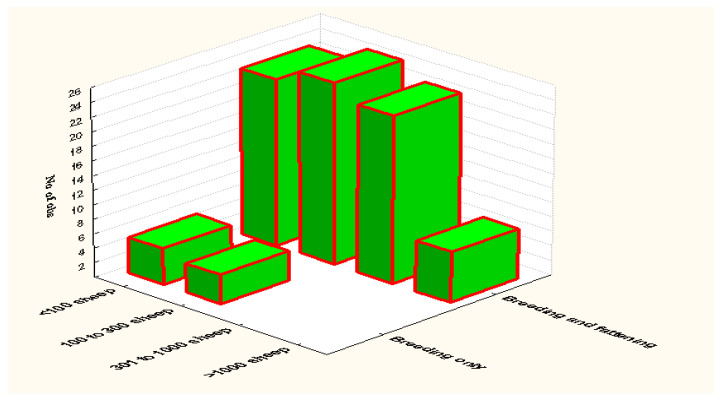
Comparison of sheep farming practices by farm size: Breeding and fattening vs. breeding only.

The results of the survey indicate that 58.62% of farmers are owner-producers, while 41.38% are both owner-producers and salaried shepherds. The majority of farmers (89.66%) use short circuits for commercialization, with only 10.34% using long circuits. In terms of mobility, 39.08% of farmers are transhumant, 36.78% are nomadic, and 24.14% are sedentary. Farm size is distributed as follows: 32.18% of farms are small (with less than 100 sheep), 33.33% are medium (100 to 300 sheep), 26.44% are large (301 to 1000 sheep), and 8.05% are very large (with more than 1000 sheep). The majority of sheep farmers engage in both breeding and fattening activities (Figure 2). Only 22.99% of farmers associate fodder production with livestock. The surveyed farms are 77.01% extensive and 22.99% are semi-extensive.

**Figure 3:**
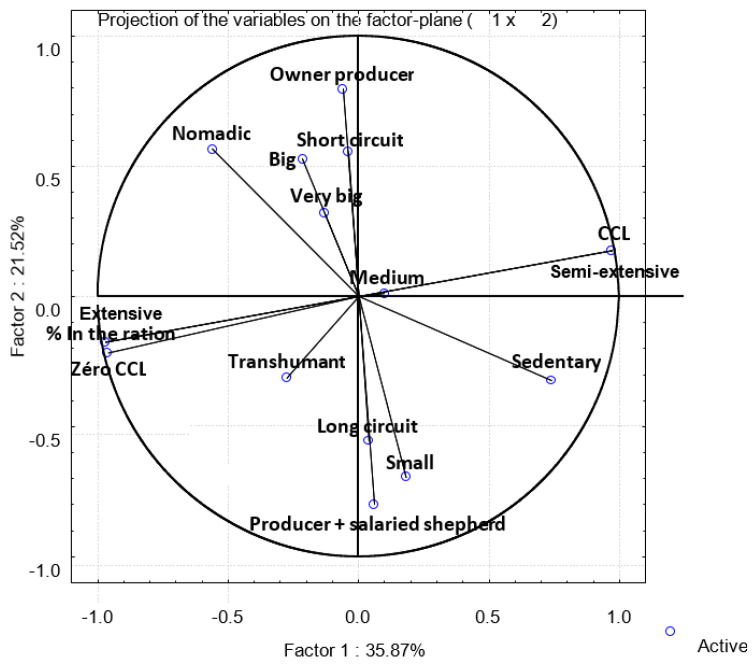
Results of the principal component analysis (PCA) of livestock farmers: variable projection.

The results of the principal component analysis show that 16 variables were included in the analysis (Figure 3). More specifically, the variables include mobility (nomadic, transhumant, and sedentary), the percentage of supplementation in the feed ration, the association of fodder crops and livestock, the extensive or semi-extensive mode, the farm size (small, medium, big, and very big), the two commercialization types (short circuit or long circuit), and the variable owner–producer or producer and salaried shepherd.

**Figure 4:**
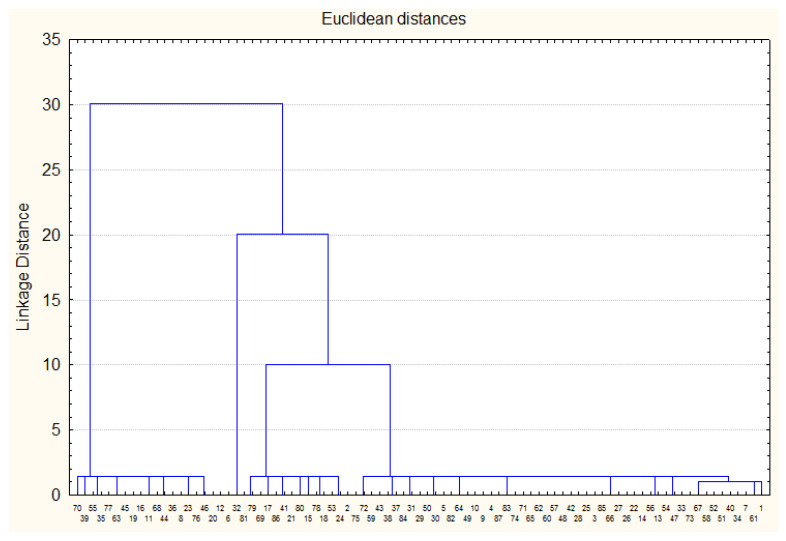
Results of hierarchical ascending classification (HAC) of livestock farmers.

**Figure 5:**
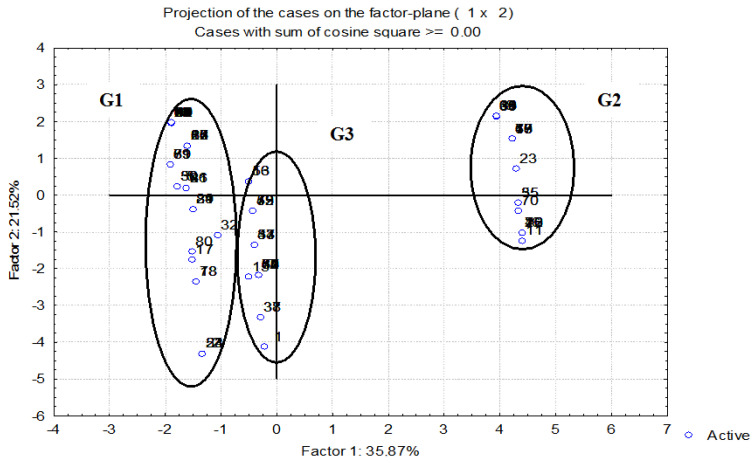
Results of the principal component analysis (PCA): a typology of surveyed livestock farmers.

The projection of data shows three different groups of livestock farms (Figure 4 and Figure 5).

The first group, including fifty-two (52) farms, was characterized by their mobility (nomadic or transhumant), with extensive herding on the steppe pastures, with supplementation by concentrated feeds that cover about 70% of the day’s feed ration.

There are two types of farmers in this first group (Table 3):

- Owner–producers: the size of their herds is often large (300 to 1000 head) to very large (over 1000 head) and more rarely medium (100 to 300 head); they use salaried shepherds to guard their livestock.
- Producers with salaried shepherds: the size of their herds is often small (less than 100 herds) to medium (from 100 to 300 herds), they guard their herds themselves, and in some cases, they also guard for other farmers for a monthly salary that varies according to the number of herds guarded. The majority of the farms in this group have adopted fattening; therefore, a short-circuit commercialization is used.

The second group includes fifteen farms, exclusively transhumant, with extensive herding. The same forms of farm managers as in the first group are also present here, but the sizes are different and the strategy of fattening is largely adopted by this group.

The third group, including 20 farms, differs from the other two groups by some characteristics, namely, sedentarization, which means that the livestock is raised in a semi-extensive system in the steppe pastures, combined with fodder production, and supplemented with concentrated feed. This group is also characterized by a relatively small to medium flock size, and there is a large majority of farms fattening their lambs, therefore the commercialization is in a short circuit. Farmers are often the owners, using salaried labor, and in some cases, the owners themselves do the work of guarding and fodder farming but never guard the herds of others.

### 3.2. Sustainability Evaluation of the Three Groups of Farms

#### 3.2.1. Agroecological Sustainability

**Figure 6:**
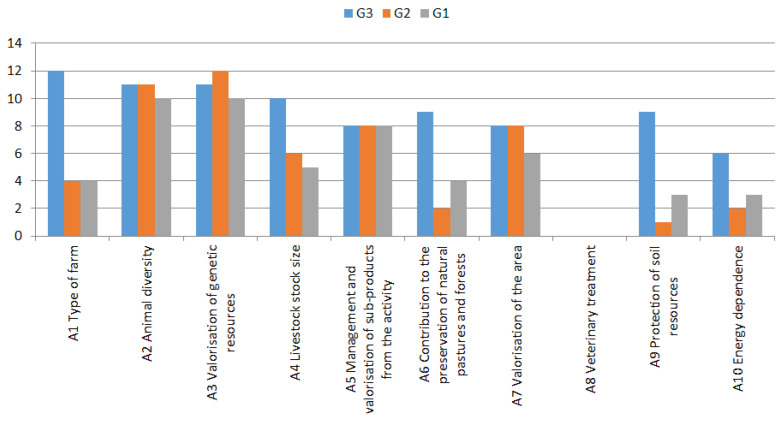
Agroecological sustainability indicators for different groups of livestock farmers.

The scores for groups G1 and G2 were average overall, but for the livestock breeding practices component, the scores were below average at 18/100 and 9/100, respectively. This is because the farms belonging to these two groups are penalized by the indicators of this component. Technical and economic choices, as well as the behavior of breeders towards the degraded state of pastures and chronic fodder deficit, along with the continuous evolution of concentrated feed prices, force them to adopt strategies to ensure the continuity of their activity. However, these strategies go against the objectives of sustainability, such as cereal cultivation without irrigation, which involves clearing already fragile pastures. The consequence of plowing such lands is an increase in their risk of degradation by erosion (water and wind), which is penalized by the indicator “A9 Protection of Soil Resources”, indicating a degradation of the soil resource (Figure 6).

Their second strategy is the seasonal movement of herds to conquer other less degraded steppe courses, which directly affects their energy consumption, especially for diesel fuel for their trucks and vans (motorization), not to mention the daily movement for watering and buying concentrates. Another strategy is the supplementation of concentrated feed in response to the fodder deficit, which is penalized by the indicator “A10 Energy Dependency”.

As a direct consequence of the fodder deficit and poor breeding conditions, farms in these two groups suffer from health problems, leading to self-medication and an overuse of veterinary products. This is demonstrated by the “A8 Veterinary Treatment” indicator. Therefore, these three indicators indicate that the livestock practices of these two groups do not promote sustainable development in the steppe environment.

In contrast, group G3 achieved an excellent score in agroecological sustainability due to good production diversity. Farms in this production group are semi-extensive sedentary farms that practice forage farming in association with livestock farming (sheep, goats, and in some cases, cattle), as indicated by the “A1 Farm Type” indicator. The “A2 Animal Diversity” and “A3 Valorization of Genetic Resources” indicators promote the diversification of livestock species and breeds in their region of origin within the farm. Furthermore, they have an excellent score for the Livestock Organization component, as demonstrated by the “A4 Livestock Stock Size” indicator, which links three characteristics: size, mobility, and feeding mode. This group has less pressure on the steppe pastures compared to groups 1 and 2, as they use cultivated forage.

For the “A5 Management and Valorization of By-Products from the Activity” indicator, group G3 obtains a score approaching the maximum, this is due to their structured use of forage cultivation. As a result, they use and valorize livestock manure to fertilize cultivated plots and also valorize wool.

In terms of the “A6 Safeguarding of the Steppe Environment” by respecting enclosures and forests, farms in this group receive the highest ratings. Their sedentary lifestyle minimizes their movement, and feeding based on cultivated forages satisfies nearly 80% of the animals’ nutritional needs.

The majority of farms in group G3, 75%, are small to medium-sized, which ensures the respect of the pastoral load. This justifies the group’s good score on the “A7 valorization of the area” indicator.

#### 3.2.2. Socio-Territorial Sustainability

**Figure 7:**
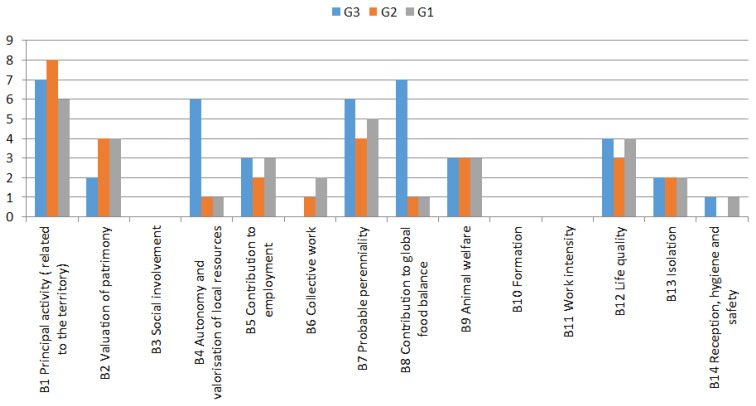
Socio-territorial sustainability indicators for different groups of livestock farmers.

For this scale, scores are rather mediocre for all three groups, with a slight positive difference in favor of G3, which is ensured by the “Employment and Services” components as well as the “Ethics and Human Development” component. However, in general, we can retain the same explanations for the three groups. All three groups are penalized by the three components of the scale. The “Quality of Products and Territories” component aims to promote the development of livestock farming that uses and respects the natural environment through appropriate practices while maintaining the authenticity of the landscape, with the aim of finding a balance between production and preservation of territories. However, groups G1, G2, and G3 are very far from achieving this expected balance. G1 scores 30/100, G2 scores 27/100, and G3 scores 36/100, because nearly 90% of all surveyed farms practice fattening in parallel with their breeding activities. This fattening activity is based on the use of concentrated feeds such as barley, corn, and soybeans, among others, which can be classified as an activity outside the soil. Consequently, this system is penalized by indicator B1, which favors local production well adapted to its natural and social environment (Figure 7).

In the same perspective, indicator B2 encourages breeders to valorize wool by weaving traditional tents, carpets, etc. This valorization of heritage not only has a preservation function but also ensures better integration of rural women in the farm economy. Unfortunately, we are witnessing a real abandonment of these traditional productions because the majority of breeders (75%) claim that they do not value wool at the household level. This is why the group’s score for B2 is poor, reaching only 16% of the planned score. For the indicator “B3 Social involvement”, not all farmers are members of associations because there are no real associations defending their cause and raising their concerns to the authorities.

The Employment and Services component promotes job creation and the provision of services to society which are not necessarily economically profitable but have a great positive impact on society, especially in rural areas. We find that the three groups are far from the objectives of this component. Taking the case of the indicator “B4 Autonomy and valorization of local resources”, we see that only 1/10, 1/10, and 6/10, respectively, for G1, G2, and G3 meet the objectives, as they depend on imported inputs for the operation and continuity of their feeding system, and at the same time, farms express a structural inability to ensure total forage self-sufficiency. For indicator “B5 Contribution to employment”, livestock farming does not require much labor, as two professional shepherds can manage a herd of 200 heads, so there is a low level of integration into the local economy and a low contribution to job creation, hence the mediocre scores obtained for this indicator.

Regarding “B6 Collective work”, according to the scores obtained, there is real individualism in the management of these farms, as each farm acts individually and seeks profit above all. We are currently witnessing the attenuation of collective work, which was one of the pillars of the agrarian society of the steppe region, especially the famous solidarity (Twiza) in work (travel, tent, plowing, etc.), and mutual aid, especially monetary (Jemla). Nowadays, this individualism reveals a major problem in the society of the region, which is the fragmentation of society and the contradiction in objectives. Instead of discussing overall development that guarantees the general interest of the region, we are witnessing a competition of individual objectives that often leads to conflicts.

In the area of ethics and human development, we can note that the profession of livestock farming is characterized by its contact and connection to the natural environment, and thus the responsibilities of the livestock farmer are increasingly important. Some of these responsibilities fall within the regulatory framework, while others are essentially moral obligations. Ethics, quality of life, personal fulfillment, and human development are intimately interdependent concepts; together they constitute essential characteristics of the social sustainability of production systems. Of the seven indicators in this component, four are almost non-existent (B8, B10, B11, B14).

For indicator “B8, Contribution to global food balance”, once again the feed problem poses a limiting factor. This indicator is evaluated by calculating the rate of imported concentrated feed relative to the size of the herd, penalizing farms that are too dependent on imported feed inputs. This is the case for the G1 and G2 farms, which are known for their total dependence on imported feed inputs.

“B10, Formation” is a fundamental condition for achieving sustainable development; it is dissemination and awareness-raising that lead farmers to understand their environment and to learn new techniques for better development, which guarantees economic profitability, social equity, and environmental sustainability. However, this constitutive parameter is almost absent for all farmers in the three groups. For “B11, Work intensity”, in general, the profession of livestock farming is characterized by the difficulty of the work, the diversification of tasks, the non-regulation of working hours, and the number of weeks of overload per year, which far exceeds the seven weeks tolerated by this indicator.

For “B14, Reception, hygiene, and safety”, we note the handling of veterinary products; unfortunately, in the majority of our sample, we found a low level of hygiene and a near absence of safety at work. In addition to that, all surveyed farmers manipulate veterinary products without seeking the advice of the veterinarian or without their presence. This is due to the sale of veterinary products to breeders despite its prohibition by law; lack of awareness about the dangers of veterinary products (overdose; interactions between products; timing, etc.); and lack of training for universal standards of hygiene and safety in the workplace.

#### 3.2.3. Economic Sustainability

We found that the G1 and G2 farms have a good average economic sustainability score of 69/100 and 64/100, respectively, which is ensured by a good balance between the components, including the “Financial Independence” component, which is a strong point for farms belonging to these groups. In fact, the “C3 Self-financing” indicator, for all surveyed farms, is self-financed, either through inheritance from generation to generation or the farmer gradually evolving in the field of livestock farming, starting as a shepherd and building up his herd over the years, eventually becoming the owner of his own farm without resorting to bank loans.

As for the “C4 Sensitivity to Aid” indicator, the vast majority of surveyed breeders do not receive state aid, and for the few beneficiaries of aid, mainly in the form of barley feed supplements, they assert that these aids are insufficient as the quantity is 400 g of barley per head per day for only 4 months a year (at most). However, according to our surveys, breeders feed their herds with 800 g to 1500 g of barley/head/day, so we see that the proposed aids are far from satisfying the nutritional needs of the herds.

**Figure 8:**
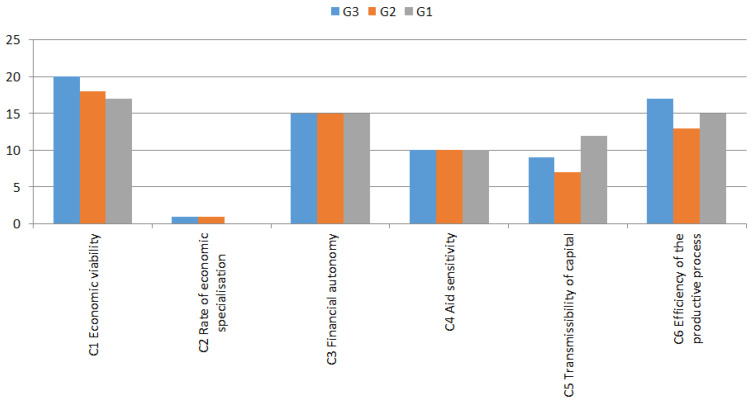
Economic sustainability indicators for different groups of livestock farmers.

However, some indicators have a very low score, such as “C2 Economic Specialization Rate”, with a score of G1 0/20, G2 1/20, and G3 1/20. The farms in G1 and G2 are characterized by a mono-production, and as a result, they suffer from the negative effects of climate disturbances and market fluctuations (Figure 8).

The farms in G3 recorded an excellent level of economic sustainability, supported by a maximum score for the “Economic Viability” component, obtained from the report of the gross surplus of the farm minus the annuities and depreciation on non-salaried work units. It was found that the farms in the second group do not have loan annuities, because these farmers do not seek bank loans and do not amortize the acquired agricultural equipment. Another factor that has a positive impact on this indicator is the reduced number of employees, which minimizes production costs, as well as the reduced number of non-salaried work units, as it is usually the owner and his eldest son. The “Financial Independence” component is also a strong support for the economic sustainability of this group, with the total self-financing of the farm through inheritance, self-progression of the owner, or a combination of both. Another favorable point for this component is the absence of state aid, which strengthens the financial independence of the farm.

The results of the sustainability evaluation are based on a maximum of one hundred (100) points, which represents the ideal farm (Table 4).

According to the rule that the overall sustainability score of a farm is the smallest score of the three dimensions, we have the scores of 32/100 for Group 1, 29/100 for Group 2, and 41/100 for Group 3.

**Figure 9:**
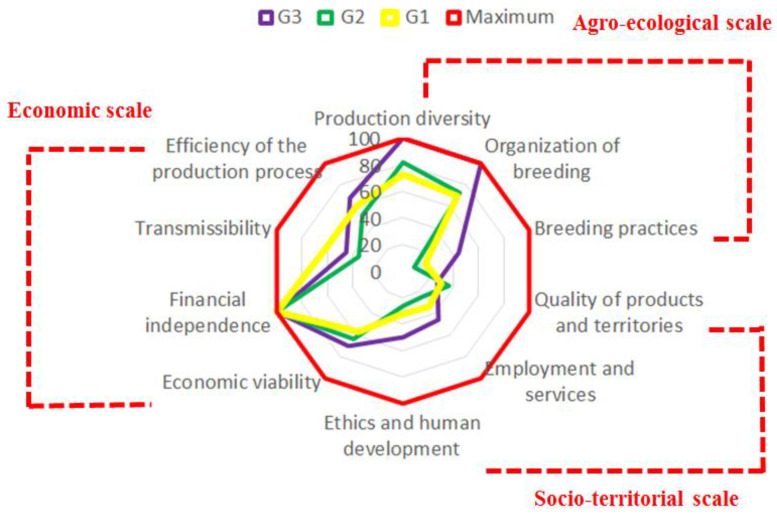
Graphical representation of the sustainability scores of different groups of livestock farmers based on agroecological, socio-territorial, and economic indicators.

The comparative graph below shows the components of each scale that influenced the scores obtained (Figure 9).

However, it is necessary to explore some of the details of the sustainability components to understand better the differences and similarities between the three groups of farms. For the agro-ecological dimension, Table 4 shows that the farms of the third group are more successful agro-ecologically with a score of 81/100 than the farms of the second group, which obtained 54/100, and the farms of the first group, which obtained 53/100. For the socio-territorial dimension, the three groups contribute to the local economy by the commercialization of their products in short circuits, but their contribution to the component “Employment and services” through job creation is not efficient, and the use of the concentrated feed does not encourage the development of livestock farming in harmony with the authenticity of the territory, which affects the component “Quality of the products and the territories”.

## 4. Discussion

### 4.1. Typology of Management Systems

Using a typology means representing the diversity of situations in the form of categories or types. This simplifies the reality by identifying some main types based on criteria considered pertinent to the studied issue. The projection of the variables on the two axes of PCA (Figure 3) shows that all variables have good correlation, and indeed these variables constitute the real characteristics of the three groups of farms resulting from the projection of the data on the two axes of the analysis (Figure 5). The first and second groups of farms are nomads: their movements are opportunist, following the pastures and water, according to an itinerary that varies from year to year depending on the availability of these resources. They are also transhumant: they make regular movements between fixed points in order to exploit the seasonal availability of pastures. Both conduct their herds in extensive mode on natural rangelands, moving to the pre-Saharan pastures in winter, which are in better condition than the steppe pastures, and to the Tellian highlands in summer for the stubble; however, today the steppe pastures represent less than 30% of the feed ration [44], supplemented by concentrates which constitute more than 70% of the feed ration per day. The third group of farms is sedentary, semi-extensive, and practices an association of forage farming and livestock, which forms the basis of their feeding systems, with a limited proportion of supplementation (<20%) in the feed ration. These aspects of feeding and mobility constitute the viability of existing management systems and are often considered as criteria of differentiation between different systems [45]. For these three groups, the major objective of the farmers is to ensure the feeding of their livestock with reasonable costs, which is essential for the success and continuity of their activity; in fact, feeding is often the keystone of livestock systems [46]. The technical criteria, including the association of livestock with agriculture, were often privileged, making it possible to differentiate between pastoral, agro-pastoral, and agricultural systems [47]. This precision allowed us to conclude that the three groups of farms in our study are part of two systems. The first system is pastoral and extensive, represented by the first and second groups, which have a feeding strategy based mainly on the movement of animals on the rangelands with supplementation of concentrates, while the second system is agropastoral and semi-extensive, represented by the third group with a feeding strategy based on forage production, the natural rangelands, and supplementation at a limited percentage.

### 4.2. Sustainability of Management Systems

The evaluation of the levels of sustainability, resulting from the typology, was conducted for the two management systems. This showed that the total sustainability scores were relatively low and under average (Table 4), indicating that there is a weakness in livestock management, far from sustainable production systems. However, these results show the following: (i) The differences between these two systems can be explained by the same system of agrarian management, pastoralism, which is based on the use of steppe pastures, and the use of concentrate, which is negatively affected by several indicators in the different components of the sustainability scale. (ii) Our results show that the real problem for both pastoral and agro-pastoral systems is the deficit of fodder, as neither supplementation nor fodder production can protect rangelands from overexploitation. Several authors [2,4,7,8,13,14,15] state that despite the increase in cultivated and purchased fodder, the rangelands offer an insufficient percentage of the total fodder needs. (iii) The study of sustainability showed that the differences in the scores between the two systems were only significant for the agroecological dimension (Table 4), and for the other two dimensions, the differences remained minor, mainly due to the grid. (iv) However, there is no doubt that the agropastoral system comes nearest to the acceptable level of sustainability (Figure 9), as integrated agriculture–livestock systems, which combine livestock and income from cultivation at the farm level, have been considered as a suitable solution for achieving a sustainable level of farming systems [48]. This agropastoralism system, based on irrigated fodder cultivation (green barley, medicago, oats, etc.), is relatively new in the steppe territory [49], and with appropriate adjustments, it will be possible to reach an acceptable level of sustainability.

To enhance the pastoral system, it has been suggested that integrating fodder culture with livestock management is crucial. However, this integration should be adapted to the spatial mobility of the livestock, and, thus, its implementation may vary regionally or nationally depending on the capacity of livestock farms for displacement. Some authors [50,51,52,53,54] propose that the integration of crop and livestock management can be organized structurally beyond the farm level, involving local groups of farmers and stock-breeders who negotiate land allocation and material exchange patterns such as manure and straw [55]. One important characteristic of pastoral breeding is their flexibility in response to environmental changes; the evaluation of this aspect assists us in orienting future interventions to promote the development of a sustainable livestock production system in steppe environments.

## 5. Conclusions

The objectives of this study were firstly to understand the different production and management systems within the pastoral system, as well as the new strategies adopted to preserve their activities in the context of the major changes affecting the steppe region. Secondly, we wanted to evaluate the sustainability of these systems in the environmental, economic, and social dimensions. The results show that there are two types of moving farms: nomadic and transhumant, which are extensive farms, representing the extensive pastoral production system, and sedentary semi-extensive farms, constituting another production system—the agropastoral semi-extensive. The study of the sustainability of these two production systems shows some similarities in the scales and differences in the final scores. The reasons for the relatively modest sustainability results can be summarized as the issue of fodder resources and their management. Suggestions for improvement of these two production systems stem from their nature, maintaining their flexibility, and proposing alternative management models, applied at various scales from the farm level to the local and regional integration, and then to the national level. The originality of our contribution is to understand the diversity of farm management and strategies in order to support decision-making in terms of models of development for each type. This study offers various research opportunities for improving the sustainability evaluation method and developing more specific indicators for the pastoral system and particularly for the steppe rangelands. Additional research should focus on alternative livestock farm strategies for solving the problem of fodder deficit, in harmony with the environment of the steppes.

## Figures and Tables

**Table 1:** Characteristics of differentiation and variables of analysis in livestock production in steppe regions.

Characteristics of Differentiation	Livestock Farmer Type	Commercialization	Mobility of the Farm	Size of the Farm	Concentrated Feed Supplementation	Practice of the Association of Fodder Production and Livestock	Intensification
**Variables of the analysis**	Owner–producer	Long circuit	Nomadic	Small	Supplementation	Yes	Extensive
	Owner–producer and salaried shepherd	Short circuit	Transhumant	Medium	Zero supplementation	No	Semi-extensive
			Extensive sedentary	Large	Percentage in feed ration (%)		
				Very large			

**Table 2:** Sustainability assessment grid for livestock production in steppe regions: indicators and scoring criteria.

Scale	Components	Indicators	Maximum Values	
Agro-ecological	Production diversity	Type of farm	12	Total maximum 33 units
		Animal diversity	12	
		Valorization of genetic resources	12	
	Organization of the area	Livestock stock size	10	Total maximum 33 units
		Management and valorization of sub-products from the activity	10	
		Contribution to the preservation of natural pastures and forests	10	
		Valorization of the area	10	
	Livestock breeding practices	Veterinary treatment	12	Total maximum 34 units
		Protection of soil resources	12	
		Energy dependence	12	
Socio-territorial	Quality of products and territory	Principal activity (related to the territory)	12	Total maximum 33 units
		Valuation of patrimony	12	
		Social involvement	12	
	Employment and services	Autonomy and valorization of local resources	10	Total maximum 33 units
		Contribution to employment	10	
		Collective work	10	
		Probable perenniality	10	
	Ethics and human development	Contribution to global food balance	10	Total maximum 34 units
		Animal welfare	3	
		Formation	6	
		Work intensity	7	
		Life quality	6	
		Isolation	3	
		Reception, hygiene, and safety	4	
Economic	Economic viability	Economic viability	20	Total maximum 30 units
		Rate of economic specialization	10	
	Independence	Financial autonomy	15	Total maximum 25 units
		Aid sensitivity	10	
	Transmissibility	Transmissibility of capital	20	Total maximum 20 units
	Efficiency	Efficiency of the productive process	25	Total maximum 25 units

**Table 3:** Characteristics of the different groups of surveyed livestock farmers.

		G1	G2	G3
	Number of Farms	52	15	20
Type of farmer	Owner–producer	31	7	13
	Owner–producer and salaried shepherd	21	8	7
Commercialization	Long circuit	3	4	2
	Short circuit	49	11	18
Mobility of the farm	Nomadic	31	1	0
	Transhumant	21	13	0
	Sedentary	0	1	20
Farm size	Small	14	7	7
	Medium	16	5	8
	Large	16	3	4
	Very large	6	0	1
Percentage of concentrated feed in the ration	Percentage	70	80	20
Association of fodder production and livestock	Yes	0	0	20
	No	52	15	0
Intensification	Extensive	52	15	0
	Semi-extensive	0	0	20

**Table 4:** Scale scores for each group of livestock farmers based on agroecological, socio-territorial, and economic indicators.

		Group 1	Group 2	Group 3
Scales	Agro-ecological	53	54	81
	Socio-territorial	32	29	41
	Economic	69	64	72

## Data Availability

Not applicable.
